# Pulmonary neuroendocrine cells in pediatric asthma and bronchopulmonary dysplasia: neuroimmune-developmental mechanisms and translational implications

**DOI:** 10.3389/fmed.2026.1898900

**Published:** 2026-08-21

**Authors:** Jianfeng Jiang, Zhiyuan Zhao, Chen Huang, Haizhen Ding

**Affiliations:** Department of Pediatrics, The Fourth Affiliated Hospital of Jiangsu University, Zhenjiang, China

**Keywords:** bronchopulmonary dysplasia, gastrin-releasing peptide, ILC2, neuroimmune regulation, pediatric asthma, pulmonary neuroendocrine cells, type 2 inflammation

## Abstract

Pediatric asthma, bronchopulmonary dysplasia (BPD), and viral-induced wheeze arise from distinct clinical settings, but all occur in the context of a maturing pediatric lung, where epithelial injury, inflammation, neural inputs, and repair or remodeling programs are closely interconnected. This intersection between pediatric lung maturation and inflammatory injury provides a rationale for focusing on pulmonary neuroendocrine cells (PNECs), developmentally enriched airway epithelial sensor cells that translate environmental and injury-related cues into local neuroimmune, repair, and remodeling responses. In pediatric asthma, PNEC-derived calcitonin gene-related peptide (CGRP) has been implicated in ILC2-associated type 2 inflammation, although CGRP–ILC2 signaling appears context-dependent and should be interpreted alongside epithelial alarmins such as IL-33, IL-25, and TSLP. PNEC-derived γ-aminobutyric acid (GABA) further links neuroendocrine signaling to IL-13-associated mucus remodeling and goblet cell responses. In neonatal lung injury and BPD, gastrin-releasing peptide and bombesin-like peptides have been linked to oxygen-associated stress, inflammatory remodeling, and impaired alveolar or vascular development; recent biomarker studies extend the relevance of this pathway to contemporary preterm lung disease and motivate further mechanistic investigation. Viral-induced wheeze is discussed as a related extension of epithelial alarmin–neuroimmune crosstalk. Overall, this review positions PNECs as cellular nodes linking pediatric lung maturation, environmental sensing, neuroimmune regulation, and epithelial repair or remodeling, with implications for mechanistic phenotyping, biomarker exploration, disease stratification, and future human-centered studies in pediatric respiratory disease.

## 1 Introduction

Pediatric respiratory diseases such as asthma, bronchopulmonary dysplasia (BPD), and early-life viral-induced wheeze arise within a biologically dynamic context in which the lung is not fully mature but undergoing continuous structural, immunological, and neurodevelopmental maturation. These disorders also impose a substantial global health burden during critical periods of lung development. Global Burden of Disease 2021 estimates indicate that approximately 126.2 million children and adolescents were living with asthma in 2021, with approximately 26.8 million incident cases worldwide; Asthma is also recognized as the most common chronic disease in children (1). Prematurity, which provides the major clinical substrate for BPD, affected an estimated 13.4 million newborns in 2020, representing more than one in ten births globally (2). Early-life viral respiratory disease is similarly substantial: global estimates indicate that respiratory syncytial virus (RSV) causes approximately 33 million acute lower respiratory infections, 3.6 million hospitalizations, and about 100,000 deaths annually among children younger than 5 years (3). These epidemiological data underscore the need to understand how developmental stage modifies epithelial injury, neuroimmune signaling, and repair across pediatric respiratory diseases. In this setting, environmental exposures and inflammatory insults are not merely triggers of disease but active modulators of developmental trajectories, influencing epithelial differentiation, immune programming, and tissue repair processes (4–6). BPD represents a prototypical disorder of disrupted lung development and remains a major chronic respiratory morbidity among extremely preterm survivors, in whom prenatal and postnatal injury interferes with alveolar and vascular growth, while pediatric asthma reflects chronic inflammatory airway remodeling occurring in a structurally evolving airway system (7, 8). Early-life viral-induced wheeze further links acute epithelial injury and immune activation to later susceptibility to chronic airway disease (9–11). Although clinically distinct, these conditions collectively highlight the broader principle that inflammatory and injury-related signals operate within a maturing lung environment, where developmental programs remain active and responsive. This developmental–inflammatory context suggests that pediatric airway disease cannot be fully understood through immune mechanisms alone, but instead requires an integrated perspective that considers how epithelial, neural, and immune systems coordinate tissue adaptation during lung growth and injury response.

Pulmonary neuroendocrine cells (PNECs) are rare but developmentally enriched airway epithelial cells that acquire specialized sensory and neurosecretory properties during early lung development. They are present as solitary epithelial cells or clustered neuroepithelial bodies (NEBs), which are preferentially located at airway branch points, positioning them within anatomically strategic microdomains where epithelial, neural, and immune components converge (12–14). These NEB-associated niches form local neuroepithelial-immune interfaces that facilitate close interactions between PNECs, airway epithelial progenitors, innate lymphoid cells, and other resident immune populations, thereby establishing spatially organized signaling hubs within the developing and injured lung (15, 16). Functionally, PNECs can be considered multimodal epithelial sensors capable of integrating environmental and injury-related cues and translating them into coordinated neuroimmune and epithelial responses, including regulation of immune cell activity, modulation of epithelial differentiation, and participation in tissue repair and remodeling processes (17). This combination of developmental enrichment, strategic localization, and multifunctional signaling capacity provides a conceptual basis for considering PNECs as key epithelial-sensory nodes within the pediatric lung, forming the foundation for the distinct neuroimmune and developmental signaling axes discussed.

Pediatric asthma, BPD, and early-life viral-induced wheeze represent distinct disease entities characterized by airway remodeling, developmental lung injury, and early-life inflammatory responses, respectively (4, 6). Despite their clinical differences, these conditions occur within a maturing lung in which epithelial injury, immune activation, and tissue remodeling are tightly coupled with ongoing developmental processes. Within this context, PNECs provide a conceptual cellular link as developmentally enriched epithelial sensory nodes that integrate environmental cues with neuroimmune signaling and epithelial repair programs. Although recent reviews have addressed PNECs in asthma pathogenesis and experimental airway models, these studies have largely focused on single disease settings or model systems, and a broader developmental framework connecting PNEC biology across pediatric asthma, BPD, and related early-life inflammatory airway disease remains limited (18, 19). In this review, we address this gap by organizing current evidence around a PNEC-centered epithelial-sensory framework, integrating three major signaling programs–the calcitonin gene-related peptide (CGRP)-related immune modulation axis, γ-aminobutyric acid (GABA)-associated epithelial remodeling pathway, and the gastrin-releasing peptide (GRP)/bombesin-like peptide (BLP) axis in neonatal lung injury–while considering viral-induced wheeze as an extension of early-life epithelial–immune interactions. We further highlight translational implications for biomarker development and disease stratification, and emphasize the importance of distinguishing well-established mechanisms from less established or hypothesis-generating pathways in pediatric respiratory disease.

## 2 PNECs as neuroendocrine sensors in the developing and injured pediatric lung

Pulmonary neuroendocrine cells represent a specialized epithelial population that sits at the intersection of airway development, environmental sensing, and local tissue regulation. They originate from airway epithelial progenitors and acquire a specialized neuroendocrine phenotype under the control of transcriptional regulators such as ASCL1 and Notch-associated pathways. ASCL1 promotes neuroendocrine fate, whereas Notch signaling restrains it during lung epithelial differentiation (12, 17, 20). In both fetal and neonatal lungs, PNECs are relatively enriched and organize either as solitary epithelial cells or as clustered NEBs located at airway branch points, creating spatial microdomains strategically positioned for local signal integration (14, 21). These NEBs are often found in close proximity to airway epithelial progenitors, group 2 innate lymphoid cells (ILC2s), and mast cells, forming local epithelial-neural-immune microenvironments that provide opportunities for paracrine and neuroendocrine crosstalk (15, 16).

Achaete-scute family bHLH transcription factor 1, Notch signaling, and oxygen sensing form a developmentally coordinated regulatory network that shapes PNEC specification, abundance, and functional plasticity. During airway development, ASCL1 is required for PNEC lineage specification, whereas Notch–HES1 signaling restrains neuroendocrine differentiation and maintains the balance between neuroendocrine and non-neuroendocrine epithelial lineages (19, 22). This developmental program remains responsive to disease-associated stimuli. In allergen-exposed mouse airways, house dust mite challenge reduced Notch signaling and promoted PNEC hyperplasia through proliferation of existing PNECs and transdifferentiation of club cells, whereas pharmacological activation of Notch limited this response (23). Following epithelial injury, Notch activation within PNECs can also promote their proliferation and conversion toward non-neuroendocrine epithelial lineages, illustrating the plasticity of this pathway during tissue repair (24). ASCL1-dependent PNEC specification has corresponding functional consequences in allergic airway inflammation, as Ascl1-mutant mice lacking PNECs show markedly reduced type 2 inflammatory responses and goblet-cell remodeling (25). Oxygen tension adds another layer of developmental regulation, with PNEC differentiation showing marked stage-dependent responsiveness during fetal lung development (26). Together, these findings connect developmental regulation of PNEC fate with disease-associated epithelial plasticity, suggesting that alterations in the ASCL1–Notch program and oxygen-responsive signaling may influence the neuroimmune, remodeling, and repair functions of PNECs during pediatric lung injury.

The developmental timing of PNEC differentiation also defines windows during which environmental or inflammatory perturbations may have different consequences. In the human lung, neuroendocrine cells are among the earliest differentiated airway epithelial populations, appearing at approximately 8 weeks of gestation, with neuroepithelial bodies detectable soon thereafter and bombesin-positive cells emerging by approximately 10 weeks (27, 28). Recent human fetal lung atlases spanning 10–19 gestational weeks and 5–22 post-conception weeks further demonstrate the temporal and spatial emergence of pulmonary neuroendocrine lineages during the pseudoglandular and early canalicular stages, when airway epithelial fate specification and proximal–distal patterning remain highly dynamic (29, 30). A second developmentally important interval extends from the late second trimester into the third trimester, when PNECs and NEBs increase in number and neuroendocrine peptide profiles shift toward peripheral airways (27, 28). This interval overlaps the canalicular-to-saccular transition in extremely preterm infants, during which immature airways, alveolar progenitors, and pulmonary vasculature are simultaneously exposed to postnatal hyperoxia, mechanical ventilation, infection, and inflammatory signals associated with BPD (8). Experimental fetal lung studies further support stage-dependent sensitivity: hypoxia markedly altered ASCL1 expression and PNEC differentiation when exposure began at an early embryonic stage, whereas later-stage fetal lung explants were considerably less responsive to the same perturbation (26). After birth, continued maturation of PNEC–neural interactions creates an additional early-life window in which allergen-induced NT4 signaling can increase PNEC innervation and GABA secretion (31). Together, these developmental transitions indicate that the biological consequences of PNEC activation depend not only on the nature of the insult but also on when it occurs relative to airway lineage specification, neuroendocrine maturation, and alveolar–vascular development.

Pulmonary neuroendocrine cells are anatomically innervated and integrated with airway neural circuits that could mediate upstream sensing of environmental and injury-related stimuli. NEBs are contacted by vagal sensory fibers and other sensory afferents, providing a structural basis for bidirectional communication between airway sensory neurons and PNECs/NEBs (32). PNECs express the mechanosensitive ion channel Piezo2, enabling them to detect mechanical stretch or airway distension and communicate with vagal afferents, which may initiate protective reflexes and influence bronchomotor tone and mucociliary clearance (33). Therefore, PNECs are positioned to integrate epithelial injury, sensory neural inputs, and local neuroimmune signals, with their integration into sympathetic and parasympathetic remodeling representing an emerging dimension of pediatric asthma and BPD biology.

The molecular repertoire of PNECs provides the basis for their downstream biological effects. Single-cell transcriptomic profiling demonstrates that PNECs express a broad array of neuropeptides, neurotransmitter-associated genes, and receptor molecules, including CGRP, GABA-related pathways, GRP/bombesin-like peptides, and 5-hydroxytryptamine (5-HT; serotonin)-related components, collectively forming a multimodal signaling repertoire that can act on immune cells, epithelial progenitors, smooth muscle, vascular cells, and distal alveolar niches (17). PNEC secretion is regulated by distinct upstream sensory pathways rather than by a uniform injury response. In human PNEC-enriched airway models, house dust mite (HDM) extract induces CALCB expression and CGRP release through protease-activated receptor-1 (PAR1); both protease inhibition and PAR1 antagonism suppress this response, whereas a PAR1 agonist reproduces it (34). Earlier studies also showed that volatile chemical stimulation of human airway PNECs can evoke CGRP release, consistent with their chemosensory phenotype (35). GABA secretion is closely linked to neural regulation during early-life allergen exposure. In neonatal mice, allergen challenge increases neurotrophin-4 (NT4), enhances PNEC innervation and nodose neuronal activity, and drives GABA hypersecretion from PNECs (31). More recently, the lysophosphatidic acid (LPA)/LPA1 pathway has emerged as another upstream regulatory signal: in a chronic HDM-induced asthma model, LPA1 localized to PNECs, and LPA1 antagonism reduced PNEC-associated GABA and CGRP production together with goblet-cell hyperplasia (36). These studies indicate that PNEC secretory control is already developmentally active in early life, with neural regulation particularly prominent for GABA and direct allergen–PAR1 sensing providing a defined upstream mechanism for CGRP secretion.

## 3 PNEC-related neuroimmune and developmental signaling programs in pediatric lung disease

**FIGURE 1:**
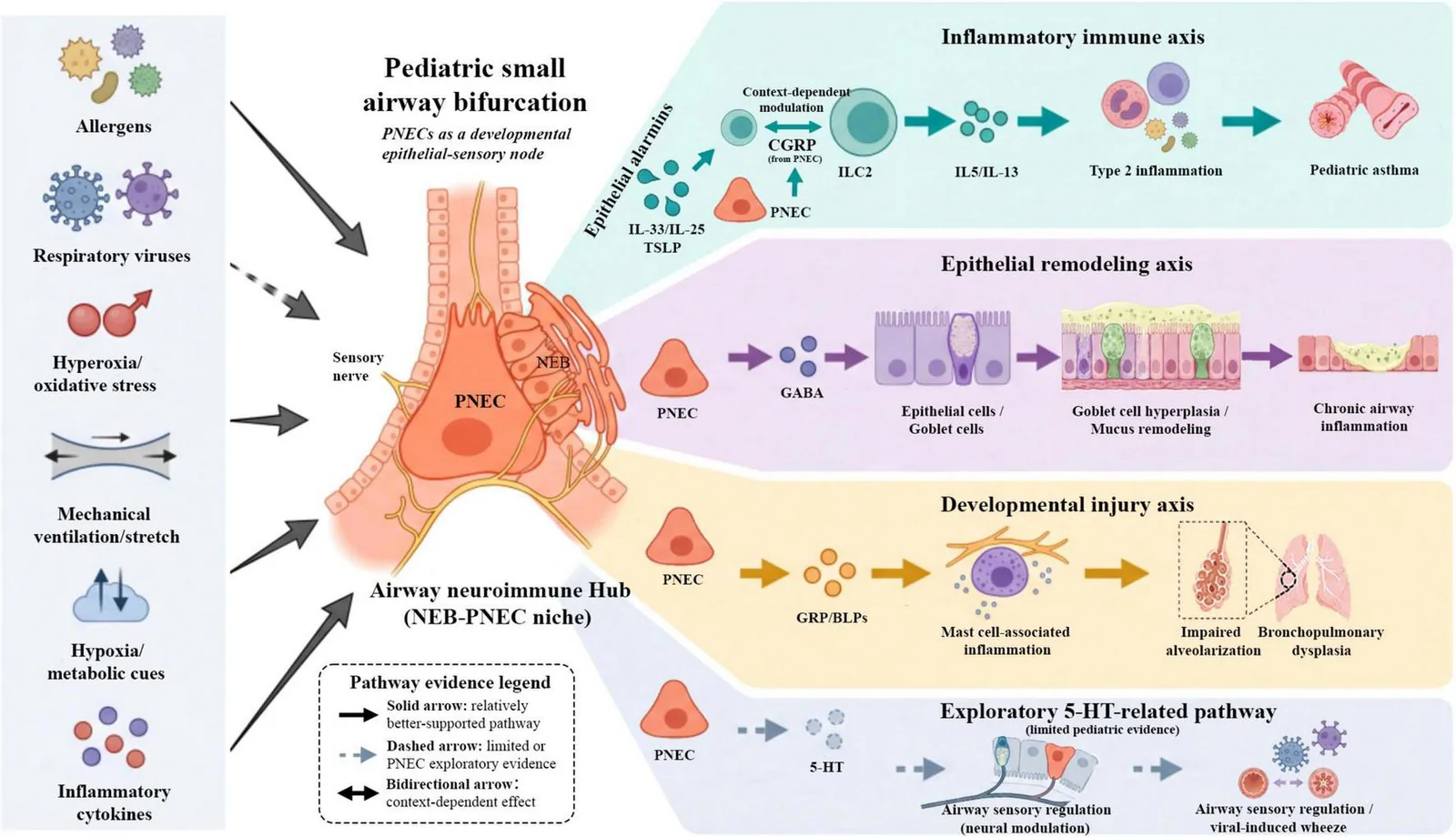
Pulmonary neuroendocrine cell (PNEC)-centered developmental epithelial-sensory framework in pediatric airway inflammation and neonatal lung injury. Environmental and injury-related cues activate PNECs within airway branch-point and neuroepithelial body niches. CGRP modulates epithelial alarmin–ILC2 responses in a context-dependent manner, GABA links PNECs to mucus remodeling, and GRP/BLPs connect neonatal injury with impaired alveolar or vascular development. The 5-HT-related route is shown as an exploratory pathway with limited pediatric evidence. Solid arrows indicate relatively well-supported pathways, dashed arrows indicate exploratory or indirect evidence (including virus-associated pathways involving PNEC-related mediators), and bidirectional symbols indicate context-dependent effects.

Pulmonary neuroendocrine cells participate in neuroimmune and epithelial signaling processes that link airway development with inflammatory and repair responses. For example, CGRP is involved in ILC2-associated type 2 inflammatory responses in asthma, GABA regulates epithelial differentiation and mucus remodeling, and GRP/bombesin-like peptides are implicated in developmental injury-related processes associated with neonatal lung injury and BPD. Based on differences in mediators, disease settings, developmental windows, and the strength of experimental and clinical evidence, these pathways are best organized as distinct PNEC-anchored regulatory axes that operate across different pediatric lung disease contexts. In addition, these signaling programs may interact with epithelial alarmin pathways, including IL-33, IL-25, and thymic stromal lymphopoietin (TSLP), in a manner dependent on developmental stage and local microenvironmental context. The following sections discuss each axis according to its biological context and the type and extent of available supporting evidence. As summarized in Figure 1, these pathways are presented as parallel PNEC-anchored signaling programs within a developmental epithelial-sensory framework.

### 3.1 PNEC-CGRP-ILC2 axis in type 2 inflammation and pediatric asthma

The CGRP-related axis represents one of the best-characterized PNEC-associated neuroimmune pathways in allergic airway inflammation, linking epithelial neuroendocrine signals with ILC2-mediated type 2 responses. In the study by Sui et al., PNECs were shown to localize near ILC2s at airway branch points, forming spatially organized epithelial–immune microdomains within the airway mucosa (25). PNEC-derived CGRP stimulated ILC2 activity and contributed to downstream type 2 immune responses in experimental allergic asthma models, whereas genetic loss of PNECs attenuated allergic airway inflammation. ILC2s are central innate immune effectors in type 2 airway disease; in response to epithelial alarmins and local tissue-derived signals, they produce IL-5 and IL-13, thereby promoting eosinophilic inflammation, airway hyperresponsiveness, goblet cell metaplasia, mucus production, and epithelial remodeling (37–39). In this setting, PNEC-derived CGRP can be viewed as a local neuroimmune signal that modulates ILC2 responses within airway branch-point niches rather than as an isolated upstream driver.

This pathway is relevant to pediatric asthma because type 2 inflammation remains one of the most clinically meaningful endotypes in children, particularly in patients with allergic sensitization, eosinophilic inflammation, recurrent exacerbations, and responsiveness to biologic therapies targeting IgE, IL-5, or IL-4/IL-13 pathways (40, 41). Recent studies further support the responsiveness of PNECs during allergic airway inflammation. Kim et al. reported that house dust mite exposure induces PNEC hyperplasia and allergen-associated PNEC heterogeneity in a mouse model of asthma, suggesting that PNECs are dynamically remodeled during allergic inflammation (23). Complementing these animal data, Mann-Nüttel et al. further defined an upstream mechanism for this response using human PNEC-enriched airway models. HDM exposure induced CALCB expression and CGRP release in a dose-dependent manner, whereas PAR1 antagonism or protease inhibition suppressed the response and direct PAR1 activation reproduced it, linking allergen protease sensing to PNEC-derived CGRP secretion (34). Human asthmatic lungs have also been reported to contain increased PNECs, complementing experimental loss-of-function evidence showing that PNEC depletion attenuates allergic airway inflammation (25). PNEC activation may also vary across pediatric asthma endotypes. Current mechanistic evidence is weighted toward T2-high allergic disease, because the principal PNEC studies have used allergen-driven models and have linked PNEC-derived CGRP and GABA to epithelial alarmins, ILC2 activation, IL-5/IL-13 signaling, eosinophilic inflammation, and mucus remodeling (23, 25, 40). This T2-associated context is clinically relevant, as longitudinal pediatric data show that T2-high asthma differs from T2-low disease in persistence, airway resistance, reversibility, and airway hyperresponsiveness (42). By contrast, non-T2 pediatric asthma encompasses neutrophilic and paucigranulocytic phenotypes with distinct inflammatory profiles, including greater representation of IL-6- and MMP9-associated responses in neutrophilic disease (43). PNECs can also respond to neural, lipid, and infection-related inputs through TRPA1-associated neural signaling, LPA/LPA1 activation, and virus-associated neuroendocrine pathways, providing candidate mechanisms through which PNEC activity could extend beyond classical T2 inflammation (36, 44, 45). The available evidence therefore supports a T2-enriched PNEC program while raising the possibility that non-T2 asthma engages a different combination of sensory and neuroendocrine inputs. Direct endotype-stratified analysis of PNEC abundance, activation state, and mediator profiles would help define whether these pathways segregate with T2-high, neutrophilic, or paucigranulocytic pediatric asthma.

CGRP–ILC2 signaling also exhibits bidirectional regulatory properties across different inflammatory contexts. Wallrapp et al. demonstrated that CGRP negatively regulates alarmin-driven type 2 cytokine production and proliferation by lung ILC2s, and that loss of the CGRP receptor component RAMP1 enhances type 2 inflammatory responses (46). Nagashima et al. further showed that CGRP shapes ILC2 function in a selective manner, supporting IL-5 production while limiting IL-13 expression and ILC2 proliferation in response to alarmins and neuropeptides (47). These findings indicate that the effect of CGRP on ILC2s is not uniformly pro-inflammatory, but depends on variables such as timing and duration of exposure, peptide concentration, ILC2 subset state, expression of the CGRP receptor complex CALCRL/RAMP1 on ILC2s, and the balance between IL-5- and IL-13-dominant outputs.

Accordingly, PNEC-derived CGRP functions within the broader epithelial alarmin network, where it can locally tune the magnitude and cytokine profile of ILC2 responses. In pediatric type 2 airway inflammation, IL-33, IL-25, and TSLP remain major epithelial signals that initiate and amplify ILC2 responses (11, 48). CGRP may refine these responses locally by tuning ILC2 activation, proliferation, and cytokine selectivity within neuroepithelial niches. This interpretation also aligns with recent work showing that neural circuits and neuropeptides influence airway hyperresponsiveness, inflammation, and symptom persistence in allergic disease (49). The PNEC–CGRP–ILC2 axis is also pharmacologically accessible. In an ovalbumin-induced allergic asthma model, the CGRP receptor antagonist rimegepant reduced airway hyperresponsiveness, inflammatory-cell infiltration, mucus-producing cell hyperplasia, and IL-5 production, together with reductions in pulmonary ILC2 numbers and IL-33-stimulated IL-5 release (50). These findings identify the CGRP receptor complex as an intervention point linking PNEC neuropeptide output with ILC2-dependent type 2 inflammation. The therapeutic effect is likely to be most relevant in airway environments in which PNEC-derived CGRP functions as an amplifier of alarmin-driven ILC2 activity, providing a rationale for phenotype-directed modulation of this neuroimmune circuit (46, 47). Thus, the PNEC–CGRP–ILC2 axis represents a well-supported but biologically flexible neuroimmune pathway in allergic airway inflammation, with its direction and magnitude shaped by the surrounding alarmin milieu, developmental stage, and local airway microenvironment.

### 3.2 PNEC-derived GABA in IL-13-associated epithelial mucus remodeling

Pulmonary neuroendocrine cells appear to regulate not only immune-cell activity but also epithelial structure and secretory differentiation. Early work by Xiang et al. established that airway epithelial GABAergic signaling, particularly GABA_*A*_ receptor-associated signaling, is functionally involved in mucus overproduction in asthma, as inhibition of GABA receptor signaling reduced allergen- or IL-13-induced goblet cell hyperplasia and mucus production in experimental models (51). The upstream control of this pathway has been particularly well characterized in early-life models. In neonatal mice, allergen exposure increased NT4 expression, promoted hyperinnervation of PNECs and nodose neuronal hyperactivity, and enhanced GABA secretion; disruption of NT4 signaling prevented these neural and secretory changes (31). This developmental nerve–PNEC mechanism was subsequently extended to infant non-human primates. Barrios et al. showed that PNECs are a major pulmonary source of GABA in mouse, non-human primate, and human lungs. In infant non-human primate asthma models, PNEC-derived GABA cooperated with IL-13 to increase MUC5AC expression and promote goblet cell hyperplasia, linking neuroendocrine secretion to epithelial mucus remodeling (52). Thus, compared with the CGRP-related immune regulatory pathway, the GABA-associated pathway is better understood as an epithelial remodeling axis centered on mucus production and goblet cell responses.

This mechanism is also biologically important because IL-13 is a central driver of airway epithelial remodeling in type 2 inflammation. IL-13 promotes goblet cell metaplasia, increases MUC5AC expression, alters ciliated cell differentiation, and contributes to mucus plugging and airflow obstruction in asthma (53, 54). Recent human airway epithelial studies further support this concept, showing that IL-13 exposure increases MUC5AC and reduces ciliated cell features in severe asthma-related epithelial models, effects that are only partially reversed by IL-4Rα blockade (55). Within this epithelial program, PNEC-derived GABA may act as a neuroendocrine co-signal that reinforces IL-13-associated mucus remodeling as part of an integrated neuroendocrine–epithelial signaling program. This interpretation also helps distinguish the GABA pathway from the CGRP–ILC2 axis: CGRP primarily links PNECs with immune-cell regulation, whereas GABA links PNECs with epithelial differentiation and secretory remodeling.

Studies have also expanded the upstream context in which PNEC-GABA signaling may operate. Recent work has further identified the LPA/LPA1 axis as an upstream regulator of PNEC activation. In a chronic HDM-driven asthma model, LPA1 was localized to PNECs, while pharmacological LPA1 blockade reduced PNEC-associated GABA and CGRP production and attenuated goblet-cell hyperplasia, connecting a bioactive lipid inflammatory signal directly with PNEC neurosecretory activity (36). Allergen-driven PNEC activation and heterogeneity have also been reported in experimental asthma, while human PNECs can release CGRP in response to house dust mite extract, supporting the broader idea that PNECs remain dynamic epithelial responders during allergic inflammation (23, 34). PNEC-associated mucus remodeling can be targeted at several levels of this pathway. Pharmacological blockade of GABA receptor signaling reverses IL-13-associated MUC5AC expression and goblet-cell proliferation in human airway epithelial models, supporting GABA signaling as a downstream intervention point for mucus-dominant asthma (52). Upstream modulation of PNEC activation provides an additional strategy: in chronic allergen-exposed mice, the LPA1 antagonist AM095 reduced PNEC-associated GABA and CGRP production and attenuated goblet-cell hyperplasia (36). More recently, disruption of the vagal TRPA1–PNEC axis reduced PNEC activation and allergic inflammation, while human airway single-nucleus transcriptomic analyses before and after bronchial thermoplasty showed attenuation of PNEC-associated NRG1–ERBB communication following treatment (45). Together, these studies outline complementary therapeutic entry points at the level of upstream PNEC activation, neuroendocrine mediator release, and downstream epithelial responses. The GABA-associated pathway may be particularly relevant to asthma phenotypes characterized by mucus plugging, chronic cough, recurrent exacerbations, and persistent epithelial secretory changes, where mucus pathology cannot be fully explained by immune-cell activation alone.

### 3.3 PNEC–GRP/BLP axis in BPD, oxygen-associated injury and impaired alveolarization

Beyond airway inflammation and epithelial mucus remodeling, PNEC signaling in the developing lung has also been linked to alveolar development, injury responses, and tissue repair. The PNEC–GRP/BLP axis is particularly relevant to BPD because it connects developmental immaturity with oxygen-associated epithelial injury. In the premature lung, disrupted alveolarization, impaired vascular development, inflammation, and oxidative stress occur during a period when PNECs are developmentally enriched and neuroendocrine signaling may still influence lung growth programs (7). GRP, the mammalian homologue of bombesin, is mainly produced by PNECs in the lung and is highly expressed during fetal development, suggesting that excessive or prolonged GRP/BLP signaling after preterm birth may disturb the transition from developmental signaling to postnatal repair (56). Hyperoxia, hypoxia, and reactive oxygen species have been proposed to induce PNEC degranulation and GRP release, providing a mechanistic link through which oxygen exposure and oxidative stress can activate PNEC-derived injury pathways in the neonatal lung (57). These oxygen-associated responses may intersect with the developmentally regulated ASCL1–Notch program described above, providing a potential link between altered PNEC differentiation, neuroendocrine signaling, and disrupted repair during premature lung development (19, 26).

Clinical and animal observations support a candidate role for GRP/BLP signaling in BPD pathogenesis. Urinary bombesin-like peptide elevation was reported to precede clinical evidence of BPD in premature infants, suggesting that BLP-related activity may appear early during disease evolution (58). Earlier studies linked BLP signaling to neuroendocrine-cell and mast-cell hyperplasia in BPD-like injury, while BLP blockade attenuated these cellular responses (59, 60). Subsequent work showed that treatment with the anti-BLP monoclonal antibody 2A11 reduced lung injury and alveolar-wall thickening, increased endothelial incorporation into secondary septa, and improved subsequent alveolarization (61). In newborn mice, bombesin administration inhibited alveolarization and promoted pulmonary fibrosis-like changes, providing experimental evidence that excessive BLP signaling can disrupt postnatal lung development (62). These findings position excessive PNEC-derived GRP/BLP signaling as a modifiable developmental-injury pathway connecting inflammatory remodeling with alveolar and vascular growth.

The biological context of GRP/BLP signaling has evolved together with the changing concept of BPD. Classic studies of this pathway were largely conducted in baboon and newborn-mouse models in which oxygen toxicity, ventilator-associated injury, inflammation, and fibroproliferative remodeling were prominent features. Contemporary “new BPD,” by comparison, is characterized predominantly by arrested alveolarization, impaired pulmonary vascular development, and dysregulated repair in extremely immature infants receiving modern neonatal care, including antenatal corticosteroids, surfactant therapy, and gentler ventilation strategies (8). Within this developmental framework, the earlier GRP/BLP findings can be integrated with current concepts of oxidative stress, inflammatory signaling, vascular growth, and alveolar maturation. The ability of BLP signaling to modify neuroendocrine-cell and mast-cell responses, alveolarization, and angiogenesis therefore provides a mechanistic bridge between classical injury models and the developmental and vascular phenotypes that characterize contemporary BPD (59–61).

More recent biomarker work suggests that the GRP/BLP axis remains relevant to contemporary preterm lung disease. Voynow et al. reported that urine GRP measured during the first postnatal week correlated with oxidative stress biomarkers and later diagnoses of BPD and post-prematurity respiratory disease in extremely preterm infants. From a translational perspective, this temporal relationship is particularly relevant because PNEC-associated activity can be detected before the later respiratory phenotype is fully established. In the multicenter cohort of extremely preterm infants, first-week urinary GRP levels above 80 pmol/mg creatinine were associated with subsequent BPD and post-prematurity respiratory disease, as well as longer durations of ventilatory and oxygen support (63). These findings support urinary GRP as a candidate early marker that may complement clinical variables and oxidative-stress biomarkers in identifying preterm infants with an evolving BPD phenotype. Overall, the PNEC–GRP/BLP axis provides a developmentally grounded link between PNECs, oxygen-associated neonatal lung injury, inflammatory remodeling, and impaired alveolar or vascular development. Future studies in contemporary preterm cohorts and human-relevant models will be important for defining how this pathway contributes to modern BPD phenotypes.

The vascular component of BPD further extends the translational relevance of PNEC biology to pediatric pulmonary hypertension. Pulmonary hypertension becomes increasingly frequent with BPD severity, with pooled incidences of approximately 5%, 18%, and 41% in mild, moderate, and severe BPD, respectively, and is associated with substantially greater mortality and respiratory morbidity in preterm infants (64). Arrested pulmonary vascular growth is now recognized as a central component of the BPD vascular phenotype, linking abnormal oxygen exposure and developmental injury to later pulmonary vascular remodeling (65). Recent experimental evidence adds a PNEC-specific mechanism to this framework: selective deletion of endothelin-2 (Edn2) in PNECs aggravated hypoxia-induced pulmonary hypertension, identifying PNECs as oxygen-responsive regulators of pulmonary vascular adaptation (66). Together, these observations provide a basis for incorporating PNEC-derived mediators into future molecular phenotyping of BPD-associated pulmonary vascular disease and for examining whether PNEC signatures identify infants with a vascular-predominant BPD phenotype.

### 3.4 Viral-induced wheeze and related pediatric contexts of epithelial–PNEC crosstalk

Early-life viral-induced wheeze represents an important pediatric context in which epithelial injury, immune activation, and airway remodeling intersect. Respiratory viral infections, particularly respiratory syncytial virus and rhinovirus, can affect the developing airway epithelium and are associated with recurrent wheeze and later asthma susceptibility in predisposed children (9, 10). Mechanistically, virus-induced epithelial injury promotes the release of epithelial alarmins, including IL-33, IL-25, and TSLP, which activate ILC2s, enhance type 2 cytokine production, and contribute to mucus remodeling and airway hyperresponsiveness (48). This places viral-induced wheeze within the same epithelial injury–alarmin–type 2 inflammatory network discussed above, while also highlighting its role as an early-life bridge between acute airway injury and later chronic airway disease.

Pulmonary neuroendocrine cell-related neuroimmune pathways may intersect with this post-viral epithelial response. Experimental influenza studies have shown that infection-induced oxidative stress can engage a GABA–GRP neuroendocrine pathway, in which GABA acts on PNECs to promote GRP release and downstream inflammatory responses. This pathway also provides a pharmacologically testable link between PNEC signaling and virus-associated lung inflammation. In influenza-infected mice, therapeutic blockade of GABA_*B*_ receptors with saclofen reduced pulmonary pro-inflammatory signaling, circulating GRP and HMGB1, and improved survival, while both GABA_*B*_-receptor antagonism and GRP antagonism reduced virus-induced monocyte infiltration into the lung (44). These findings position the GABA–GRP circuit as a pharmacologically accessible neuroendocrine pathway in virus-associated inflammatory lung injury and provide a mechanistic framework for testing PNEC-directed strategies in human airway models. Within the broader context of early-life viral airway injury, these influenza studies demonstrate that respiratory infection can activate neuroendocrine signaling circuits involving PNEC-associated mediators. In this setting, CGRP may influence alarmin-driven ILC2 responses, GABA may contribute to mucus-related epithelial remodeling, and GRP/BLP signaling may link epithelial injury with inflammatory amplification. Broader studies of airway neuroimmune regulation further support the concept that epithelial cells, sensory nerves, neuropeptides, and immune cells form integrated response networks during airway inflammation and infection (67).

Other pediatric conditions, including neuroendocrine cell hyperplasia of infancy (NEHI) and cystic fibrosis, have also been associated with PNEC or neuroepithelial body abnormalities. NEHI is a PNEC-hyperplasia–dominant childhood interstitial lung disease, whereas cystic fibrosis has historically been associated with altered bombesin-, calcitonin-, and serotonin-immunoreactive PNECs but is primarily driven by cystic fibrosis transmembrane conductance regulator dysfunction, mucus obstruction, chronic infection, and neutrophilic inflammation (18). These conditions are therefore considered briefly as related pediatric contexts rather than primary disease anchors for the present developmental–inflammatory framework. Overall, viral-induced wheeze provides a clinically relevant extension for studying how early epithelial injury, alarmin release, PNEC-associated mediators, and neuroimmune regulation may interact during pediatric airway disease.

## 4 Translational implications: biomarker development, disease stratification, and therapeutic considerations

At the translational level, PNEC-related candidates can be broadly divided into functional mediators and cellular identity markers. CGRP, GABA, and GRP/BLPs are functional mediators whose interpretation depends on the disease and developmental context discussed above, whereas chromogranin A (CHGA), synaptophysin (SYP), and achaete-scute family bHLH transcription factor 1 (ASCL1) are better regarded as PNEC-enriched histological or transcriptional markers for tissue-based studies (17, 68). Therefore, future biomarker studies should distinguish between markers of PNEC presence, markers of PNEC activation, and mediators with plausible pathogenic activity. Table 1 synthesizes these candidates by distinguishing functional mediators from cellular identity markers and by summarizing their disease contexts, evidence limitations, and translational interpretation.

**TABLE 1:** Pulmonary neuroendocrine cell (PNEC)-related mediators and markers in pediatric respiratory disease.

Mediator	Main biological role	Disease relevance	Evidence position	Representative references
CGRP	Modulates ILC2 activation, proliferation, and IL-5/IL-13 output within epithelial alarmin-driven type 2 inflammation	Pediatric asthma; allergic airway inflammation	Well-characterized but context-dependent neuroimmune pathway	Sui et al. (25); Wallrapp et al. (46); Nagashima et al. (47); Thio and Chang, (37); Kim et al. (23); Mann-Nüttel et al. (34)
GABA	Cooperates with IL-13 to promote MUC5AC expression, goblet cell hyperplasia, and mucus remodeling	Asthma with mucus remodeling; chronic pediatric airway inflammation	Supported but context-dependent epithelial remodeling pathway	Xiang et al. (51); Barrios et al. (52); Boomer et al. (55)
GRP/BLPs	Associated with mast cell responses, inflammatory remodeling, and impaired alveolar or vascular development	BPD; oxygen-associated neonatal lung injury; post-prematurity respiratory disease	Developmental-injury pathway supported by experimental and biomarker evidence	Sunday et al. (60); Cullen et al. (58); Ashour et al. (62); Subramaniam et al. (61); Voynow et al. (63)
5-HT/serotonin-related signaling	May participate in airway sensory signaling, epithelial-neural communication, and bronchomotor regulation	Possible relevance to airway reactivity and post-viral airway responses	Exploratory pathway with limited pediatric disease-specific evidence	Noguchi et al. (12); Kuo et al. (17); Thakur et al. (15)
CHGA/SYP	Neuroendocrine granule and vesicle markers used to identify PNECs/NEBs	Tissue-based assessment of PNEC abundance or hyperplasia	Useful histological markers, not disease-specific biomarkers	Cutz et al. (21); Cutz et al. (16); Noguchi et al. (12)
ASCL1	Key transcription factor for PNEC lineage specification and neuroendocrine differentiation	Developmental PNEC biology; altered epithelial differentiation after injury	Key developmental regulator; mainly tissue- or omics-based marker	Eenjes et al. (22); Noguchi et al. (12); Kuo et al. (17)
EDN2	Regulates oxygen-responsive pulmonary vascular adaptation and modulates vascular responses to hypoxia	BPD-associated pulmonary hypertension; hypoxia-related pulmonary vascular remodeling	Emerging PNEC-specific vascular regulatory pathway	Suzuki et al. (66)

Functional mediators and cellular identity markers are summarized according to their biological roles, disease contexts, evidence positions, and translational interpretation. CGRP is presented as a context-dependent modulator within the epithelial alarmin–ILC2 network, GRP/BLPs as a developmental-injury pathway linking inflammatory remodeling with alveolar and vascular development, EDN2 as an emerging PNEC-specific regulator of pulmonary vascular adaptation, and 5-HT-related signaling as an exploratory pathway. CHGA, SYP, and ASCL1 are included as tissue or lineage markers rather than disease-specific soluble biomarkers.

The translational value of PNEC-related pathways may differ according to disease phenotype and developmental stage. In BPD, urinary GRP provides a non-invasive readout of early neuroendocrine activation and may be integrated with oxidative-stress markers and clinical variables to identify infants with an evolving respiratory disease phenotype (63). In BPD-associated pulmonary hypertension, the convergence of arrested vascular development, oxygen-sensitive PNEC signaling, and emerging PNEC-derived vascular mediators such as EDN2 provides an additional molecular dimension for cardiopulmonary phenotyping (64–66). In virus-associated lung injury, the GABA–GRP pathway offers both measurable neuroendocrine mediators and pharmacologically accessible signaling nodes, as experimental antagonism of GABA_*B*_ or GRP signaling attenuates inflammatory responses after influenza infection (44). In pediatric asthma, CGRP- and GABA-linked PNEC activity may help refine endotype-level phenotyping, particularly by distinguishing a T2-enriched PNEC–ILC2/mucus-remodeling program from PNEC responses driven predominantly by neural, lipid, or infection-associated inputs in non-T2 inflammatory settings. This disease-oriented framework allows PNEC-related markers to be evaluated not simply as measures of cell abundance, but as indicators of specific neuroimmune, epithelial, or vascular programs.

Pulmonary neuroendocrine cell-related pathways provide several levels of therapeutic intervention that differ across pediatric respiratory phenotypes. In type 2-high allergic asthma, modulation of the PNEC–CGRP–ILC2 interface offers a direct neuroimmune strategy, supported experimentally by CGRP receptor antagonism that suppresses ILC2 activation and multiple asthma phenotypes (50). Mucus-dominant disease provides a complementary epithelial target through inhibition of PNEC-derived GABA signaling or its upstream regulators, including the LPA/LPA1 and nerve–PNEC pathways (31, 36, 45, 52). In the premature lung, the therapeutic architecture is distinct: modulation of GRP/BLP signaling has improved alveolarization and vascular-associated structural outcomes in experimental BPD, placing the PNEC–GRP/BLP axis within developmental lung-repair strategies (61). These disease-specific pathways support a therapeutic framework in which PNECs can be approached through upstream sensory activation, mediator release, receptor signaling, or PNEC interactions with immune and epithelial target cells. For CGRP-directed intervention, the surrounding alarmin environment and the balance of IL-5- and IL-13-dominant ILC2 responses may help define the most responsive inflammatory context, given the selective and context-dependent effects of CGRP on ILC2 function (46, 47).

More broadly, therapeutic targeting of PNEC-related neuropeptide or neurotransmitter pathways in children is closely linked to developmental context. CGRP, GABA, GRP/BLPs and related signaling systems participate in normal lung development, airway sensation, epithelial repair, and tissue homeostasis (67). This concern is especially important in preterm infants and young children, where developmental window, dose, reversibility, off-target effects, long-term pulmonary outcomes, and neurodevelopmental safety are central considerations. Progress toward translation will therefore depend on pediatric tissue studies, longitudinal cohorts, spatial omics, and human PNEC-based experimental models that can clarify when PNEC-associated mediators serve as biomarkers of injury, contributors to disease mechanisms, or potential targets for developmental-stage- and phenotype-specific intervention strategies.

## 5 Literature-search approach and current limitations

### 5.1 Literature-search approach

This review was developed as a narrative synthesis rather than a systematic review or formal evidence-grading study. Relevant literature was identified through searches of PubMed, Web of Science, Scopus, and Google Scholar from database inception to July 2026, using combinations of terms including “pulmonary neuroendocrine cells,” “neuroepithelial bodies,” “PNEC,” “pediatric asthma,” “bronchopulmonary dysplasia,” “viral-induced wheeze,” “CGRP,” “GABA,” “GRP,” “bombesin-like peptide,” “ILC2,” and “epithelial alarmins.” We prioritized mechanistic studies, human pediatric or neonatal data, relevant animal and non-human primate models, and recent reviews addressing PNEC biology, neuroimmune regulation, airway inflammation, or neonatal lung injury. Studies focused primarily on pulmonary neuroendocrine tumors or adult non-pediatric lung disease were not included unless they provided directly relevant biological context.

### 5.2 Current limitations of the evidence base

Within this narrative framework, the current evidence base for PNEC-related pediatric lung disease remains uneven across diseases, species, and experimental systems. The CGRP- and GABA-related pathways in allergic airway inflammation are supported by mechanistic animal studies, selected human models, and associative human observations. Experimental loss- and gain-of-function studies further show that PNECs and their mediators can actively shape disease phenotypes: genetic depletion of PNECs attenuates allergic airway inflammation, whereas BLP blockade reduces neonatal lung injury and exogenous bombesin disrupts alveolarization and promotes remodeling (25, 60–62). The GRP/BLP axis in BPD is derived predominantly from baboon and newborn-mouse models, complemented by clinical biomarker studies linking early urinary BLP/GRP signals with subsequent respiratory outcomes (58, 63). Viral-induced wheeze is supported mainly by epithelial alarmin biology and virus-related neuroimmune models rather than direct pediatric PNEC studies (44). These differences emphasize the importance of defining disease- and developmental-stage-specific PNEC programs rather than assuming a uniform neuroendocrine mechanism across pediatric respiratory disorders.

Several methodological features further shape the current field. PNECs are rare and spatially clustered, making their recovery by conventional dissociation-based approaches variable and increasing the importance of tissue architecture when interpreting cell–cell interactions (17, 19). Access to pediatric lung tissue is inherently restricted, particularly for longitudinal sampling, and most human evidence therefore comes from small tissue series, airway models, biomarker cohorts, or rare-disease cohorts rather than serial tissue-based studies. Differences among mouse, non-human primate, and human PNECs in developmental stage, innervation, peptide expression, and airway organization also complicate direct cross-species comparison. In addition, PNEC abundance, activation state, and mediator release are not yet assessed through standardized experimental or clinical readouts, while endotype-stratified pediatric studies remain uncommon. These features make longitudinal human sampling, spatially resolved analysis, and standardized measurement of PNEC-related mediators central priorities for the next phase of the field.

## 6 Long-term consequences and future directions

### 6.1 Long-term consequences of altered PNEC function

Altered PNEC activity during early lung development may have consequences that extend beyond the initiating injury by influencing neural–epithelial communication, mucus differentiation, inflammatory responsiveness, and alveolar–vascular maturation. Neuroendocrine cell hyperplasia of infancy (NEHI) provides a clinically informative example of a persistent PNEC-associated phenotype. In the French FRENCHI cohort, children with NEHI followed for a mean of 68 months showed heterogeneous respiratory trajectories, including recurrent wheezy exacerbations, later asthma diagnoses, and persistent obstructive abnormalities in a subset of patients (69). A larger European multicenter study of 378 children with persistent tachypnea of infancy/NEHI similarly found progressive improvement in many patients but persistent hypoxemia, exercise intolerance, imaging abnormalities, or pulmonary-function impairment extending into adolescence in a subset (70). These longitudinal observations provide a clinical context in which sustained alteration of the pulmonary neuroendocrine compartment is associated with respiratory phenotypes that can persist well beyond infancy.

In asthma, an additional long-term dimension may arise from early remodeling of the PNEC–neural interface. Early-life allergen exposure increases NT4-dependent PNEC innervation, neuronal activity, and GABA secretion, linking developmental neural plasticity with mucus remodeling (31). The greater persistence and airway hyperresponsiveness observed in T2-high childhood asthma further provide a rationale for determining whether sustained PNEC–ILC2 or PNEC–GABA activity tracks persistent wheeze, mucus-dominant disease, or altered airway responsiveness across childhood (42).

In the premature lung, altered PNEC signaling occurs during a period in which alveolar and pulmonary vascular development remain highly active. Experimental manipulation of GRP/BLP signaling can modify alveolarization, fibrosis-like remodeling, and angiogenesis during neonatal lung development (61, 62), while PNEC-derived EDN2 has recently been linked to hypoxia-associated pulmonary vascular adaptation (66). This developmental context is relevant to the long-term respiratory trajectory of children born preterm, in whom altered lung-function trajectories can persist from childhood toward adult life (71). Longitudinal integration of PNEC-related mediators with lung function, airway reactivity, imaging, pulmonary vascular phenotypes, and respiratory morbidity could therefore determine whether early neuroendocrine alterations identify specific trajectories of later lung disease.

### 6.2 Human-centered and spatial approaches

Future progress will depend first on better human sampling strategies and spatially resolved approaches. Longitudinal pediatric asthma cohorts and contemporary preterm infant cohorts are needed to link PNEC-related mediators with disease trajectories rather than single time-point phenotypes. In BPD, minimally invasive airway samples, including tracheal aspirates, bronchoalveolar lavage, nasopharyngeal aspirates, and post-mortem infant lung tissue, may help connect epithelial injury, immune activation, and developmental remodeling. Recent patient-derived airway models and single-cell studies of newborn and preterm lungs provide a framework for interrogating rare PNEC populations and their ligand–receptor interactions in human-relevant settings (72–74). Spatially resolved approaches can extend single-cell sequencing by restoring the anatomical information that is lost during tissue dissociation. Single-cell RNA sequencing is well suited to defining PNEC transcriptional states and candidate ligand–receptor programs, whereas spatial transcriptomics can determine where these rare cells reside, which cell populations surround them, and whether their molecular states vary according to airway region, developmental stage, or local injury environment. This distinction is particularly relevant for PNECs because they cluster within neuroepithelial bodies and airway branch-point niches and communicate with nerves, ILC2s, mast cells, and neighboring epithelial cells over short spatial distances. Recent spatial profiling of human lung tissue has demonstrated that rare neuroendocrine epithelial cells can be mapped according to their proximal–distal distribution and local cellular neighborhoods (75). In asthma, single-cell spatial transcriptomics has further identified discrete pro-inflammatory airway niches characterized by specific cellular proximities, chemokine and alarmin expression, and remodeling patterns that are not apparent from cell-type composition alone (76). Similarly, spatial transcriptomic analysis of developing and injured neonatal human lungs has resolved age- and injury-associated cellular programs within their native tissue architecture, providing a framework for studying developmental lung disease (77). Applied to PNEC biology, these approaches could distinguish PNEC–ILC2, PNEC–mast cell, PNEC–nerve, and PNEC–epithelial microdomains and relate local CGRP, GABA, or GRP-associated signaling to specific sites of inflammation or remodeling. Spatial proteomic and multiplex-imaging approaches would add a complementary protein-level layer by mapping neuropeptides, receptors, epithelial alarmins, and immune-cell markers within the same tissue neighborhoods.

### 6.3 Experimental models and causal validation

Disease-relevant human experimental models offer a direct route for testing causal relationships. Human PNEC-enriched airway models, air–liquid interface (ALI) cultures, airway organoids, and stem-cell-derived respiratory epithelial systems can be used to test whether allergens, hyperoxia, viral infection, oxidative stress, or mechanical stretch directly activate human PNECs and induce CGRP, GABA, or GRP/BLP release (78, 79). Recent advances in human induced pluripotent stem cell (iPSC)-derived PNECs, ALI differentiation systems, and airway organoid models provide practical platforms for studying rare epithelial cell populations and virus–epithelial interactions under controlled conditions (80, 81). Co-culture systems will be particularly useful for testing PNEC–ILC2 regulation of type 2 cytokine responses, PNEC–epithelial interactions in GABA–IL-13-associated mucus remodeling, and PNEC–mast cell or PNEC–mesenchymal crosstalk in GRP/BLP-associated neonatal lung injury. Across all settings, future translation will require careful attention to developmental timing, dose, reversibility, off-target effects, and long-term pulmonary and neurodevelopmental safety.

Across these experimental and clinical settings, longitudinal study designs will be particularly informative when serial PNEC-related measurements are linked to developmental stage, pulmonary function, imaging, exacerbation burden, oxygen requirement, pulmonary vascular status, and later respiratory outcomes. Such integration would allow early PNEC states to be connected with subsequent disease trajectories and would provide a more direct framework for identifying developmental windows and phenotypes most responsive to pathway-specific intervention.

## 7 Conclusion

Current evidence supports several distinct PNEC-related signaling programs rather than a single shared mechanism across pediatric lung diseases. Accordingly, the field has moved from viewing PNECs primarily as rare neuroendocrine epithelial cells toward considering them as epithelial sensory nodes that participate in disease-specific inflammatory, epithelial remodeling, and developmental injury responses within the maturing pediatric lung. Their translational value currently lies mainly in mechanistic phenotyping, biomarker exploration, and disease stratification. Future studies using pediatric samples, longitudinal cohorts, spatial omics, and human PNEC-based models will help define causal relationships and identify developmental stages and disease phenotypes most amenable to PNEC-directed translation.
